# Interactive numerals

**DOI:** 10.1098/rsos.160903

**Published:** 2017-04-26

**Authors:** Harold Thimbleby, Paul Cairns

**Affiliations:** 1 Department of Computer Science, University of Swansea, Wales SA2 8PP, UK; 2 Department of Computer Science, University of York, York YO1 5DD, UK

**Keywords:** interactive system, numbers and numerals, human error

## Abstract

Although Arabic numerals (like ‘2016’ and ‘3.14’) are ubiquitous, we show that in interactive computer applications they are often misleading and surprisingly unreliable. We introduce **interactive numerals** as a new concept and show, like Roman numerals and Arabic numerals, interactive numerals introduce another way of using and thinking about numbers. Properly understanding interactive numerals is essential for all computer applications that involve numerical data entered by users, including finance, medicine, aviation and science.

## 1. Introduction

This paper introduces the new concept of **interactive numerals**. Interactive numerals highlight a wide range of hitherto unnoticed issues with entering and using numbers reliably with interactive computer systems. The reach of the concept is very broad, covering calculators, spreadsheets, medical devices, computerized forms, web sites, airplane cockpits—in fact, all forms of numerical data entry system.

Interactive numerals highlight common avoidable defects in many interactive systems where people use numbers. Incorrectly implemented interactive numerals cause subtle and sometimes critical problems. A proper understanding of interactive numerals should help researchers identify related problems and seek suitable solutions, as well as help practitioners recognize design problems and to decide on effective responses to problems: whether re-implementing systems, training users, upgrading or replacing or even banning the systems.

Understanding interactive numerals makes error management more reliable: deciding what to do after an error has occurred requires understanding the root causes of the error. In particular, until interactive numerals are properly implemented, using data logs in investigations of alleged user error is unreliable (devices tend to record what they do, not what the user tells them to do).

This paper shows that poorly implemented interactive numerals are surprisingly widespread; even systems developed by the world’s leading programmers are not immune. In hospitals—to take just one example of a critical application of interactive numerals—clinicians routinely enter drug doses and radiation doses into interactive systems, where numerical errors can be fatal and are often treated as criminal. It is, therefore, important to properly understand interactive numerals and any limitations they have in current systems. Indeed, this paper shows that many numerical errors are caused, not by users, but by the poor design of the systems. We will present some worrying examples taken from a wide range of systems in the body of the paper.

The implication is that interactive numerals are not well understood; this paper aims to correct that. This paper provides an analysis that should help everyone appreciate the causes of the problems and the limitations of current interactive systems. The aim is to begin to move towards more robust implementations of numbers in all digital devices, systems and services.

## 2. Numbers and numerals

We take it for granted that 2016 and 3.14 are numbers, but strictly they are *numerals*, ways of printing or writing number values. When we see 2016, we may think of a particular number, but what we see is not the number but strictly speaking it is just various ink patterns that we interpret to represent a numerical value. More specifically, the four ink patterns ‘2016’ make up an Arabic numeral, just like ‘two thousand and sixteen’ makes up an English numeral, and MMXVI is a Roman numeral. Note that the same number can be represented by many different numerals, and even by many different Arabic numerals (thus 003.14 is equal to 3.14, though 3.1400 is generally not considered exactly the same as the number represented by 3.14 because it is more precise).

We now think of Roman numerals as being rather awkward. For example, the Romans had no sensible way of writing 3.14, and Roman numerals seriously limited how people could use and think about numbers. Nevertheless, after Fibonacci introduced Arabic numerals to Europe around 1200 [1], it still took centuries for Arabic numerals to become more popular: people were attached to the familiar, and anyway many people used counting boards and other devices for everyday arithmetic.

Today we are facing a new transition, we argue as dramatic as the move away from Roman numerals to Arabic numerals. We call the new way of thinking about numbers **interactive numerals**.

As well as popularizing decimal positional notation, Arabic numerals transformed arithmetic by introducing the cipher, an explicit symbol for zero. Arabic numerals, however, cannot distinguish between no number and zero, as ‘no number’ has no valid Arabic representation. It turns out that this and many other subtle problems are rife in interactive computer systems—and often cause problems that are hard for users to understand or avoid. Interactive numerals help us think clearly about such issues, and—properly implemented—directly address such issues, as we will show in this paper.

Arabic numerals, which we are now very familiar with, are written down usually on paper or displayed on screens and *then* we read them as numbers. Interactive numerals look much the same but, unlike Arabic numerals which are only read and used *after* being written, interactive numerals are read and processed by a computer that has to represent them (at least on the screen if nowhere else) *as* they are being written. Thus interactive numerals consider the process of writing down a numeral, corrections and all, not just its final form as a full-fledged numeral. As a special case, interactive numerals handle the case of no numeral yet written. By contrast, nothing yet written is impossible to represent correctly as an Arabic numeral as it has no digits.

Imagine typing the number −0.5 into a computer; unlike the traditional piece of paper, the computer has to make sense of each and all of the intermediate steps as we type 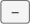, then 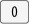 then 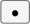 and so on. This raises many subtle problems. For example, – itself is not a number at all, and −0 is strictly equal to 0. Yet the computer has to decide how to represent the interactive numeral at every step, even before the user has finished entering it. In many cases, as we will show, the computer makes premature decisions about the representation that also change the meaning of what is being entered.

Interactive numerals occur everywhere users enter numeric data into computers. Put very briefly, the problem is that not all interactive numerals are Arabic numerals, but assuming they are—which is a common conceptual error—leads to problems and use errors that are very hard to recognize, let alone correct. Indeed, in our early work [2], where we noticed there was a problem but had not fully grasped its extent, we did not make a sharp distinction between numerals and numbers, and we fell victim to some confusions ourselves. The new contribution of this paper, then, is not just bemoaning the problem, but providing a formal framework wherein problems can be identified and solutions can be worked out.

Unfortunately, as we will show, problems with interactive numerals are widespread. When mathematicians, programmers and scientists notice and can reason about these problems and hence seek solutions, every user (and their work) will benefit—even if they do not notice any changes.

## 3. Definitions

### 3.1. Numeral

Numerals are sequences of symbols that follow agreed conventions for interpreting them as number values. In this paper, we consider digit-based systems of numerals for representing numbers rather than, say, counters or linguistic word-based numerals like English phrases.

Given a set of symbols *S*, generally called *digits*, a **numeral** is a non-empty string of symbols *S*^+^ together with a surjective function N that maps each string to a numeric value. Typically, N can be expressed explicitly in a simple arithmetical way. Note that special symbols, such as *π*∈*S*, may or may not be considered numerals depending on the context.


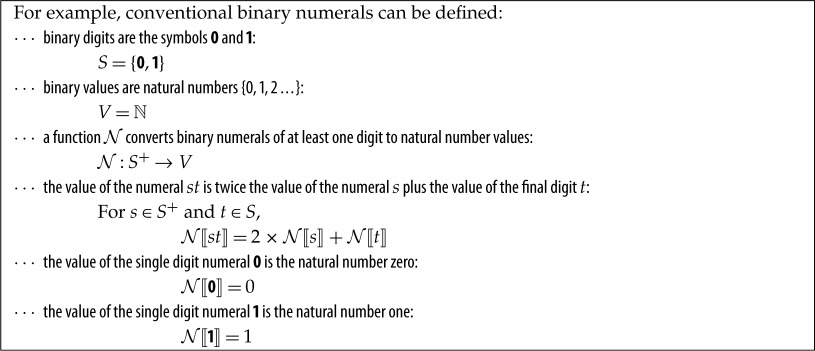


There are trivial generalizations so *S* can include signs (+, −), radix points^1^ and digit block separators (e.g. the space or comma), and of course generalizing to other bases than 2 is trivial. Further notation may be used to represent precision, choice of base and so on [3].


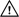
 Since N defines an equivalence class, if for two numerals *x*,*y* we have N⟦x⟧=N⟦y⟧ we tend to say *x* equals *y* or even that *x* is *y*. It is therefore common and very easy, and frequently harmless, to confuse numerals and numbers.

Now, depending on how N is implemented (programmed) from its specification above, it can interpret a numeral like 11.1 in different ways:

$$N\text{⟦}11.1\text{⟧}=\left\{ \begin{array}{l} 1\text{ (by stopping at the radix point, reading from the right)}\\ 3\text{ (by stopping at the radix point, reading from the left)}\\ 7\text{ (by ignoring the radix point)}\\ 9\text{ (by using `subtract ASCII code of 0' to get values of symbols)}\\ \text{guarded so it cannot occur}\\ \text{undefined}\\ \text{report an exception}\\ \text{excluded by type checking at compile time, since}\;S\;\text{cannot contain}‘.^{\prime } \end{array}\right.$$

but in no case will it have the ‘obvious’ value of 3.5_10_ (11.1 in binary is 3.5 in decimal), at least if sensibly refined from our explicit specification above. This is obviously something of a toy example, but it highlights that any implementation of evaluation on a computer has to make explicit things so the computer can do them that are not necessarily explicit in the specification.

One fundamental implementation problem that is unavoidable is that computers, being finite, *cannot* implement the natural numbers, N (let alone the real numbers, R, etc), correctly. At the very least, any implementation of numerals must decide how to limit the infinite and, if done well, how to minimize and manage the impact of the ensuing errors. (Using arbitrary precision numbers would obviously help.) Implementations that neglect the impact of such fundamental problems are, as we will see, likely to cause severe problems.

### 3.2. Interactive numeral

The definition of numeral, above, takes a numeral to be a fixed entity that denotes a numerical value. By contrast, an **interactive numeral** *includes* the process intended to create a numeral. In other words, an interactive numeral additionally specifies the procedural implementation of a numeral, in contrast with the conventional purely declarative specification of an ordinary numeral, which abstracts away from the process of construction.

An **interactive numeral** may therefore be defined by a vector of string buffers *b*_*i*_∈*B*, an initial contents *b*_0_=*d* (sometimes called the ‘default,’ possibly the empty string), a set of actions A:B×N→B to modify buffers, i.e. the action *a* on buffer *b*_*i*_ gives *b*_*i*+1_=*a*(*b*_*i*_,*i*), together with a surjective function $N_{\text{int}}$ to map buffer strings to realizable numeric values *V* ∪*E* including exception and other conditions *E*. Buffers generally have associated invariants, for instance on their length, e.g. ∀*i*:0≤|*b*_*i*_|≤8.

An implementation will usually optimize buffers into a single object, such as a string, and update it on each action rather than creating a new buffer each time. In many incorrect implementations of interactive numerals, buffers are optimized directly to a numeric value, which of course cannot correctly represent exceptions (such as entering too many digits or numerals with as-yet incorrect syntax).

For example, pressing the digit **1** is an action that will create a buffer *b*_*i*+1_=*b*_*i*_**1** by appending **1** to the previous buffer *b*_*i*_. However, if *b*_*i*_ is too long, then **1** cannot be appended, and some exception condition will be flagged (or be ignored or be handled inappropriately) and typically we will have *b*_*i*+1_=*b*_*i*_ (for some *i*). Interactive numerals may additionally having timed actions, so for instance if there is a timeout (e.g. when ‘no other action occurs for 1 minute’), then typically *b*_*i*+1_=*d*.

A realizable value is an implementable representation of a numeric value; for example, for a cash machine (ATM) it may be 10n,n∈N:1≤n≤25; or it may be an IEEE 754-2008 floating point number, with or without rounding and exceptions. By contrast, for a conventional numeral, the range of valid numerals and values are rarely if ever explicitly specified. While IEEE floating point has peculiar properties that may ‘leak’ into the user interface (we give examples below), arbitrary precision reals do not avoid problems as the buffer invariants cannot be broken, and in any case buffers are not required to be real numerals (e.g. because of syntax errors).

Often there are conventional actions, such as an action 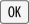
∈*A* to ‘submit’ a buffer (and perhaps leave it unchanged), an action to clear a buffer 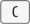
(*b*_*i*_,*i*)=*d*, and so on. When actions can include mouse movement, 
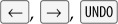
 and 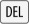 the buffer is generally refined to include pointers or stacks. In any case, note that *A* must include *every* possible action, not just a small set of conventional digits—the set *A* has to cover the possibility that the user can do anything. If the user presses a non-numeral like the space bar, what should happen? In particular, we cannot rely on type checking (as might forbid the example of N⟦11.1⟧ above) because every input is valid—type checking does not control what a user does.

In short, an interactive numeral includes how the numeral is dynamically created, giving it a continual interpretation, including for all its intermediate representations and exception conditions. In contrast with numerals, then, $N_{\text{int}}$ for interactive numerals is complex—its definition, even without the algorithm, is far more complex than a conventional numeral. We therefore do not show a specification of $N_{\text{int}}$ here.


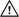
 Since numerals ‘are’ numbers in common thinking (see note in §3.1 above), it is very easy to turn a blind eye to the daunting complexity of $N_{\text{int}}$, considering it instead to be effectively N, and therefore to gloss over considering all the exception cases. This is a tempting category error, and one with consequences this paper explores.

### 3.3. Quasi-numerals

Strings of symbols that look like numerals are frequently used in contexts that might be considered more strictly to be names or identifiers. We call these **quasi-numerals**.

Bank account numbers and book numbers (ISBNs) are examples of quasi-numerals. Despite commonly being called numbers, arithmetical operations (such as doubling or adding one) make little sense with such numbers. In other respects, however, they behave very like interactive numerals. They are entered as digits and have a structure, and the value denoted must fall in a particular range to be valid. In fact, the range of correct values may be highly constrained by hidden arithmetic operations that specify a check digit as part of the numeral. (Incorrect check digits should cause exceptions.)

But bank account ‘numbers’ do not denote members of a set of conventional numeric values like N, despite being countable. Similarly, many strings of symbols that are not just digits, such as passwords or people’s names and social security numbers, are used in a similar sense of denoting members of sets. The decision of what is a digit and what is not within any character code is an arbitrary convention (e.g. is 0 a digit or a capital letter?), so inevitably the boundary between numerals and general strings is blurred.

The user interfaces that permit **quasi-numerals** (bank account numbers, car registration numbers, personal ID numbers and so on) to be created are very similar to the user interfaces that permit interactive numerals to be created. Sometimes, and confusingly, the user interfaces are identical. Therefore, there is considerable and interesting overlap in design issues across the various types of numeral and quasi-numeral. In this paper, however, we are particularly concerned where, somehow, the user interface must preserve relevant arithmetical properties of the numbers the interactive numerals are taken to represent. There is a large area of concern where the natural conception—the user’s natural conception—of N should be very simple but in fact confusingly fails to be so.

### 3.4. What interactive numerals are not

Interactive numerals arise in many areas and applications, from web pages, spreadsheets, to handheld calculators, as well as in special purpose instrumentation, burglar alarms, medical devices and more. However, it is important not to confuse interactive numerals for the applications that use them. In many areas the applications blur the distinctions, which plays into our natural tendency to equate numerals and numbers.

Spreadsheets make the problems particularly acute. A cell in a spreadsheet can certainly allow the user to enter an interactive numeral. However, the number value it finally denotes depends on the formats and formulae in its and other cells. For example, if the numeral ‘5.5 kg’ is entered in Microsoft Excel, if it is subject to the operation SUM it has value 0, but subject to PRODUCT it has value 1.^2^ Furthermore, if the numeral entered is ‘1.’ it will typically be displayed as exactly 1, without a decimal point. These interactions are complex to explain in adequate detail, and can be confusing traps for both spreadsheet implementors and users.

Another area of confusion is with calculators, which come as basic calculators (left-to-right operator precedence), so-called ‘algebraic’ calculators that respect conventional operator precedence, and reverse Polish notation (RPN) calculators. In all forms of calculator the issues of interactive numeral are very similar; however, the main differences between types of calculator are in arithmetic expression parsing—which is independent of interactive numeral treatment. In particular, while RPN calculators are popular among certain communities, they have serious problems that interfere with their otherwise consistent arithmetic parsing: the RPN stack is generally limited to a small size, and stack overflow has undefined effects [4]. Such complex and invisible problems, in our opinion, eclipse their alleged benefits.

### 3.5. Other forms of numeral

There are many generalized types of numeral, ‘things that denote number values’—such as dice, dials, increase/decrease chevron buttons, speech, clocks, counters, etc. [5], as well as names (such as month names denoting month numbers)—but the focus of this paper is on the dominant case of left-to-right sequential typing, canonically typing and entering conventional Arabic numerals to specify numeric values for interactive devices, computer programs or other applications.

We note that **sensors** may be considered a broad generalization of numeral for when physical or other processes rather than humans define numeric values. Clearly, to measure a temperature, either a thermal sensor can be directly connected to a computer, or a human can use a conventional thermometer and enter a numeral denoting the temperature that has been ‘read off’ numerals shown on the thermometer. Sensors, however, often employ numeric transforms such as low pass filters to improve measurement dependability.

## 4. Problems with interactive numerals

### 4.1. Ubiquitous examples from calculators

One of the best inventions of computers is the delete key, which allows us to correct mistakes. Suppose you want to enter 0.5 but you mistakenly enter 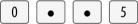, with two decimal points. You will want to correct this error. But try this on almost any calculator, and you will find that the calculator has already ‘corrected’ your error: it has decided you entered 0.5 before you have even started correcting it. Ironically, that means if you do correct the error, you will make things worse, perhaps entering 5 instead. The computer decided what you were entering before you had finished entering it; it ignored the second decimal point, which then made correcting it counter-productive.

The computer (or, rather, the computer’s programmer) wants to ensure syntactically correct numerals so they can always be represented as simple number values along with allowing you to correct them with delete. Specifically, the computer is programmed to display a valid number because that is how the value is represented internally (e.g. as a floating point number). But numbers cannot represent all interactive numerals: for example, nothing with two decimal points has any number value to represent it, but it can be corrected to be a valid numeral. It follows that trying to do both leads to unreliable and inconsistent behaviour. This is a problem, one of ‘premature semantics’: computers generally try to treat the user’s errors as valid numbers too soon when no sensible numerical meaning can or should be assigned to them at that point. Premature semantics is explained more fully below, in §5.1.


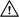
 It may be argued that a user might have entered 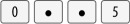 intentionally, for instance because they know how the user interface works, and pressing decimal point twice increases the chance it has been correctly pressed at least once. If so, what is the problem? The problem is that the programmer has prematurely committed the user’s probable error to be a valid number, and the error and its consequences are now unknown to the user. The programmer does not know what a user intends, and an error is usually indicative of a failure to carry out an action as intended. For example, the user may have intended to enter 0.05, but because the 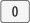 and 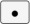 keys are close, the 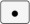 key was pressed accidentally. *The programmer has no idea*. Throwing away information on an assumption is dangerous, and avoidable.

**Figure 1.**
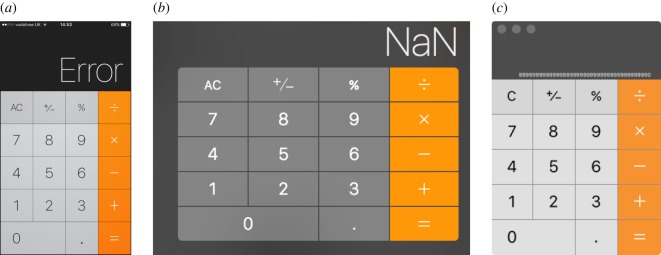
Three contemporaneous calculators, all the latest versions as of August 2016. As explained in the paper: (*a*) the error in the iOS calculator occurs when the user tries to enter 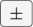 under some circumstances when it displays −0; it ought to display 0, not Error. (*b*) NaN occurs when the user tries to enter 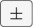 under some circumstances when the MacOS calculator displays 0; it ought to display −0. (*c*) A 40 digit number entered by the user and displayed in the MacOS app calculator is impossible to read, being about 1 mm high on the original screen. The calculator displays only some of the *least* significant digits of the number, so the display is misleading even when it can be read. (*a*) Apple iOS, (*b*) Apple MacOS pane and (*c*) Apple MacOS app.

If you set out to enter −3 on the Apple MacOS v. 10.11.6 calculator, the key sequence 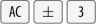 leads to the result of 3 (cf. figure 1).^3^ The change sign is probably ignored because −0 did not seem meaningful to the designer: −0 is equal to 0, which is what is displayed, so the change sign key had no effect!

**Figure 2.**
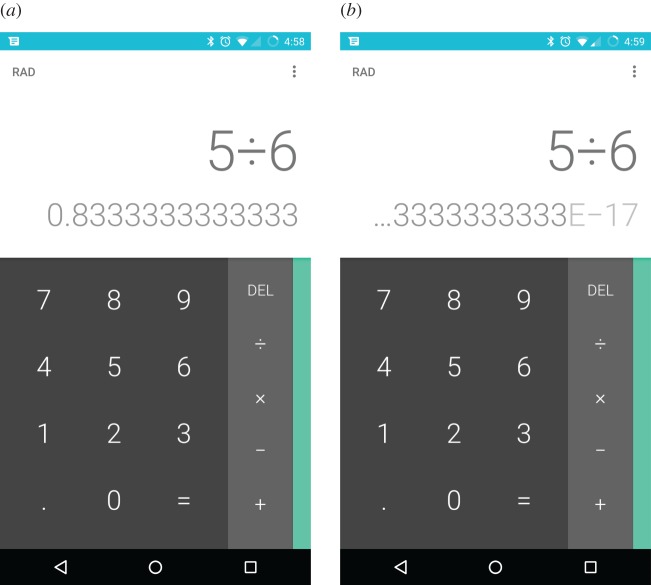
Google’s Android calculator calculating $\frac{5}{6}=0.8333\ldots$. In the right-hand image, scrolling the answer left/right produces a bizarre multiplier (E−17, presumably meaning ×10^−17^), and a hidden decimal position (off the left of the screen and out of sight), thus resulting in a meaningless result. (Compare with the Apple calculator’s use of reduced font size shown in figure 1*c*; neither approach works correctly for the user.) Screenshots provided by Martin Atkins.

And if you correct some mistakes, say typing 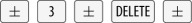 you get NaN (on MacOS), which means *Not a Number*. NaN is an internal value representation (e.g. defined in IEEE floating point standards) that means nothing to non-technical users—clearly, displaying it to general users is a fault in the calculator itself: it has ignored an error, and its number representation code has failed to display it as either a number or as an error message.

Typing 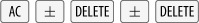 crashes Apple’s iOS v. 9.3.3 calculator (the effect of 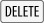 on iOS is achieved by swiping the finger left/right across the numeric display). But the same sequence displays 0 on both Apple’s MacOS calculators (the stand-alone app and the notification pane calculator).

Keying 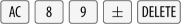 gives −8 (MacOS pane calculator) or –89 (MacOS app calculator). The key 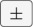 is handled differently too: on iOS, 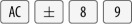 gives −89 but on MacOS it gives 89 on both calculators, but 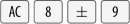 gives either −89 or 9.

So three modern calculators from a leading manufacturer that are clearly designed to look the same, which all have the same ‘look and feel’, and therefore would be expected to be identical in behaviour, all implement the same actions differently—and all behave incorrectly by any standard interpretation. Arguably they should be consistent, even if we disagree about what should be correct behaviour.

Evidently, handling interactive numerals properly is problematic. It is surprising that calculators, which have had their current form for over 40 years, are still implemented inconsistently and incorrectly. Defects of this sort are rife in all data entry devices, not just calculators.

Cut and paste, like delete, is a basic feature of modern computers: in particular, typing text and pasting text typed elsewhere should be interchangeable. The Apple calculators, however, respond to pasting differently. If you type 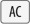 then 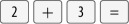, all calculators will display 5 correctly. If instead you paste 2+3=, the calculators respond differently: iOS and MacOS pane give 2, but the MacOS app calculator gives 23 (though it gives 2 if 2–3 is pasted, so + and − are treated differently). None report an error or give any indication that keystrokes are being adjusted or discarded. But cut and paste can be done correctly: in the Windows 7 calculator v.6.1, for instance, pasting ‘2+3=’ produces a 5 in the display.

To give another example from Apple’s calculators: typing 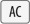 9999999999999…, a number almost as large as you like, will display as *exactly* 999,999,999 (that is, nine digits on the iOS and pane calculators) and then doing 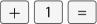 will display 1e9 (i.e. meaning 10^9^) which is wrong as the correct answer is much larger. However, on the Apple MacOS app calculator, as larger numbers are entered they get displayed in progressively smaller and smaller fonts, and finally become so small they are completely unreadable. Then pressing 
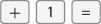
, as before, the large number is presented in a clear full-size 1E43 (or whatever). In other words, the calculators *can* display large numbers, but not when the user enters them directly!

Note that E and e are different notations, and in fact *neither* are the standard mathematical notation. In standard notation, 1E43 or 1e43 should be written 10^43^ or more generally, a number like 2.3E43 (or 2.3e43) would be written 2.3×10^43^, which all of the Apple calculators, with their high-resolution displays, are technically able to display.

Curiously, when the iOS calculator is turned sideways (to make it landscape) it becomes a scientific calculator and the user *can then* enter large numbers directly—except using a key labelled 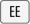, not 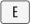, one speculates because having two very similarly named keys, namely 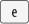 (the base of natural logarithms) and 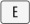 (raising to a power of ten), would have been thought to be too confusing. We would argue, then, if the key names would be confusing, this is another reason not to use the non-standard notation E (or e) at all in numerals!^4^

Although we have used Apple for the concrete examples above, other leading manufacturers, such as Casio and Microsoft, are not immune to these problems. It seems that before clarifying the concept of interactive numerals (as we do in the present paper) or equivalent, nobody thought about these details, probably as numeric data entry seems trivial and does not require much thought to *seem* to get working. But interactive numerals are not obvious and there has been a failure to learn and employ rigorous computational thinking throughout design—apparently nobody considered it necessary! The result is that there is no standard approach. Swapping one calculator for another will give different results for exactly the same calculation, and (as we have shown elsewhere) there are also many other bugs in calculators [4]. It is dangerous.

We note that people use calculators primarily because they do not know or are not certain of the answers, so bugs in calculators are very hard for them to spot and work around.


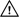
 Though these details may sound like nit-picking, they have real effects. For example, two students narrowly avoided death after an incorrect calculation performed on a mobile phone [6].

Doing a calculation more than once, and *in a different way* remains good advice, not just because it helps protect against user error but it also helps protect against design error.

### 4.2. Medical examples

**Figure 3.**
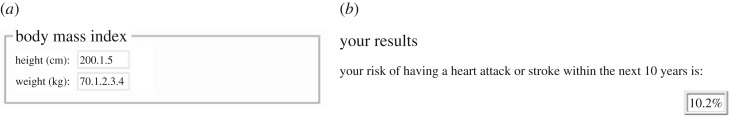
Screen shots from QRISK2, a clinically-validated risk calculator, available at www.qrisk.org. (*a*) Invalid numerals entered as data; (*b*) apparently valid result. Note that no errors are reported by QRISK2, so the user may be unaware of any problems, and then will act inappropriately. These screen shots were taken in October 2016.

We have elsewhere given examples of medical devices with interactive numeral problems [7–11] too. Another example, shown in figure 3, is taken from a ‘clinically validated risk calculator’. Evidently, clinical validation is not sufficient to check for the sorts of interactive numeral errors that are shown in the figure—which of course could undermine any clinical value of using the calculator.

**Figure 4.**
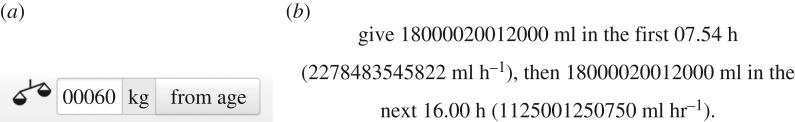
Screen shots from Mersey Burns, a multi-award winning app. The app is available from merseyburns.com. (*a*) Weight of patient in kilograms: apparently 60 kg is displayed; (*b*) calculated dose, from screen shot: the huge dose is clearly incorrect. In this example, no errors are reported by Mersey Burns so the user may act inappropriately from the advice displayed. Here, the calculated dose looks very obviously wrong (the first 7.54 h dose is over 10^9^ l h^−1^, compared with an average adult blood capacity of 5 l): evidently the app does no or inadequate validation and sanity checking. More generally, less obvious errors may be missed by clinicians using this app, with potentially harmful effects. Note that Mersey Burns is used for treating burns victims—who may be in considerable pain, and whose treatment will be urgent—so what may seem obvious to us calmly reading this paper may be easily misinterpreted under real clinical conditions. Screen shots taken in November 2016.

Screen shots from an award-winning app are shown in figure 4. The figure shows the problem of interactive numerals getting too large for their display regions; what is apparently displayed as a patient weight of 60 kg is much larger, but the value is too large to fit in the small display. The app should not allow numbers to overflow their input fields, because they are ambiguous when they do. The result will be confusion, hopefully noticed by the user and before potential patient harm.

It is interesting that despite the obvious programming problems, as illustrated in the figure, the app not only won prizes but is well known for being the first British ‘CE marked’ medical app. CE marking is a European legal mark, here meaning that an app is approved for clinical use in Europe, so evidently the CE approval process failed in this case to adequately check interactive numeral processing. We know many other problematic medical devices that have CE marks [7–12], so this implies the CE marking process is flawed as it overlooks serious design defects that may adversely affect patients.

### 4.3. Banking example

**Figure 5.**
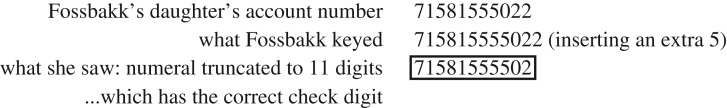
Fossbakk used her bank’s user interface to transfer money to her daughter’s account. Unfortunately she double-pressed a key, resulting in a longer account number. The user interface truncated the number, thus obtaining another, but valid, account number. Thinking she had keyed the correct number, Fossbakk confirmed the transfer, despite it being to a differently-named account, which apparently was not displayed, checked or confirmed. Details taken from Olsen [13].

In 2007, Greta Fossbakk lost 500 000 Norwegian Krone (equivalent to about US$100 000) due to an interactive quasi-numeral bug, a problem that still persists in many bank systems [10,13]. Fossbakk keyed in an extra digit in an account code that, despite making it an invalid account number, was not caught by the online banking system (figure 5). Her bank argued that she could not prove that she keyed 12 digits—as with other user interfaces this paper discusses, it seems to be no coincidence that the user interface does not log what the user does, which is to the bank’s advantage. Olsen’s [13] experiments with users entering bank account details suggest that 0.2% of all interactive bank transactions will suffer from a similar (unnecessarily) undetected error: given the scale of internet banking, this is a huge unnoticed problem.

Apart from not silently truncating numbers, the problem would also be reduced if numerals are displayed with separators (spaces, dashes, etc.), breaking the digits up into groups thus making them easier to read and, indeed, easier to type correctly. Thus, because the computer system failed her, Fossbakk alone had to notice the difference between 71581555022 and 71581555502; this would have been much easier if the numerals had been displayed as 715-815-555-502 and 715-815-555-022, which are more obviously different. Indeed, it is concerning that credit card numbers are usually displayed with account numbers split into chunks, but user interfaces typically delete the gaps thus deliberately making the numerals much harder to read, type and check.

Problems with interactive numerals are widespread, and users like Fossbakk are often penalized for design failings. We suspect that manufacturers are in denial for a combination of reasons: they do not explore the issues because of an unfortunate interpretation of product liability (they do not want to discover any problems that may be their fault) and by assuming their ‘competent’ programmers would never make mistakes over something so ‘trivial’. And of course, logging exactly what users do and exactly how systems respond to them would expose manufacturers to the truth, which may work against them in court.

### 4.4. IP numbers

IP numbers (both IPv4 and IPv6) are made up from blocks of digits. IP version 6 numbers are blocks of four hexadecimal digits separated by colons. Each block can have leading zeros suppressed, so 0012 and 12, for instance, would be treated as identical.

In 2011, Nigel Lang was arrested for downloading illegal material onto a PC owned by his wife. The police had added an extra digit onto somebody else’s IP number, obtained his wife’s physical address, then assumed he had committed an offence (males are more likely to commit internet offences). His arrest and subsequent stigma ruined Lang’s family life. Fortunately, his efforts to defend his innocence eventually resulted in a settlement of £60 000 from the police for making the errors [14], that had made him and his family suffer considerably.

It is alarming that a single digit error in a user interface can have such unchecked consequences—as well as taking over 6 years to resolve. If nothing else, the design of IP numbers (strictly, IP numerals) and IP user interfaces used by the police (and others) shows little maturity about human factors, such as using check digits (which are common on ISBNs, bank account numbers, etc.).

### 4.5. Forms

Forms often include interactive numerals. In addition to problems with individual interactive numerals (which are generally as discussed above), forms often impose constraints on the relations between some or all numerical values they allow, as well as how they can be entered.

For example, dates may be entered on a form as a triple of numerals: the day number, the month number and the year number. If the user edits the date 12/30/17 (representing 30 December 2017), say, to 2/28/17 (perhaps starting by deleting the first 1 in 12/30/17) there are possible intermediate values that are not valid dates (such as 30 February)—yet the user interface must permit editing to allow the date to be corrected. Many (bad) forms force the user to make a circuitous edit so the date is *always* valid; if so, this is a case of premature semantics, which we discuss more generally in §5.1 below.

We note that, as with many interactive numerals, it is rarely possible for a user to save a form before it is valid. For a single interactive numeral, this may be a useful precaution as ‘saving’ and ‘acting on’ a single value are closely related and perhaps too easily confused, but with a form the user may be inhibited from saving many values (such as names and addresses); in this case, the imposition of premature semantics seems to us to be a serious step backwards from paper forms, which of course save the user’s work continually regardless of its validity.

### 4.6. Discussion

Although the examples come from diverse contexts, the problems can fall into one of two types: problems of construction and problems of representation. Interactive numerals are constructed by the user but as they are being constructed they are also being interpreted by the system, at the very least so they can be represented in the display. People, though, make mistakes and so in the process of constructing interactive numerals they can be expected both to make mistakes and to try to correct those mistakes. Mistakes are also made by programmers—typically in unconsciously assuming that the input of numbers is trivial.

In the case of Fossbakk, the mistake was not detected by the computer even though it could have been. In the calculator examples, unlike bank accounts, it is not possible to know what numbers or sorts of numbers are intended by users, but it should be expected that users will attempt to correct mistakes they notice. However, as we showed, the use of standard features like using the delete key, which is supposed to help can, ironically, lead to confusion.

These are serious problems. In our experiments [15,16], we have found that users regularly enter numbers incorrectly and nearly 4% of the time they do not notice. (If you think this rate is high, then that is because you are not noticing your own errors, and you mistakenly think the rate should therefore be lower.) Some novel styles of entering numbers can reduce the error rate to experimentally undetectable levels [17].

We note that standard texts on human factors and error (e.g. Reason’s classic error taxonomy [18]) rarely discuss *noticing* error, and hence rarely note the important role that computers can have in helping users notice (and hence correct or manage) numerical and other errors, particularly because computers could in principle help notice errors and, in particular, help notice many sorts of errors humans are poor at noticing (such as errors in check digits). Insidious cases like those discussed in Moore [19] about radiotherapy computer systems show that numerical and calculation errors caused by mixes of poor programming and innocent use error can persist unnoticed for decades, and hence affect thousands of people.

Other common methods of construction, such as cut and paste, fail to alert the user to interpretations of what was constructed that deviate substantially from what was pasted.

The problems of representation are unavoidable for interactive numerals because the numbers must be represented and are unbounded, but displays are finite. Either a display truncates the interactive numerals leading to incorrect values or attempts to display them leading to the problems of unreadable displays as seen in both calculators and medical apps. The representations also fail to match with existing mathematical notations and moreover do not allow users to use such notations in a consistent way.

The conclusion from these various examples must be that this is not simply manufacturer sloppiness so much as interactive numerals are deceptively different to standard Arabic numerals; they are a newly identified and surprisingly complex phenomenon.

Indeed, realizing that interacting with numbers is complex and a serious design challenge [2] (issues we now recognize as falling within the scope of interactive numerals), we have sought and found various ways of reducing the error rate [20,21]—ways that would be invaluable in critical applications such as finance, avionics and medicine. The ability to reduce error rates with improved approaches to interactive numerals proves that current accepted ways of entering numbers are unnecessarily unreliable.

The science of interactive numerals (and other classes of user interface design issues) has not been extensively studied, particularly in the HCI (human–computer interaction) field, which one might feel would be its natural home. By contrast, say, to the widespread activity in security [22], this lack of attention seems remarkable (CHI+MED is an exception [23]). Why? Although the final outcomes of security problems and safety problems may be broadly indistinguishable, there are interesting cultural and economic differences. For security, there are many outsiders who are known to be bad, and therefore defending against them is *expected*; investment in security (and hence investment in security research) makes sense. By contrast, for safety, there are insiders who are expected and often required to be professional; blaming the user is then an easy option as the user has apparently failed to do a good job—they are the proverbial bad apple. For safety, then, it often seems the user has failed rather than the system. Hence little investment or research is demanded for user interface safety.

Since poor interactive numeral design induces use error, we hope accident and other investigators will consider it as a possible cause of incidents, and thus we hope this paper will stimulate new avenues of critical investigation and research that will reduce error and its consequences.

## 5. Analysis

It has long been recognized that computers do not do normal arithmetic. Thus, normal addition satisfies the associative rule, *a*+(*b*+*c*)=(*a*+*b*)+*c*, but on a computer this rule fails because of rounding errors. For example, 0.1 as a decimal number is a recurring binary fraction, so it cannot be represented as a simple binary value precisely.^5^

The field of numerical analysis concerns itself with such error analysis, so-called ill-conditioning and related issues [3]. Good programmers are therefore very careful to do arithmetic in ways that take numerical problems into account. Some calculators do arithmetic in base 10 (e.g. using binary coded decimal, BCD), so that users are not surprised by decimal–binary conversions causing unexpected rounding.

The corresponding problems of interactive numerals have only just been recognized (they are systematically reported here for the first time), but they are no less serious, as our examples above show. Unfortunately there has been nothing like ‘a numerical analysis’ for interactive numerals, so we aim to address that.

The problems of interactive numerals fall into the following categories:

### 5.1. Premature semantics

Premature semantics is an idea we identify in this paper for the first time.

For a computer program to interpret the user’s data entry, it must impose some semantics. For example, it may interpret numerals as floating point numbers; this semantics is very easy to implement and means that as a user enters a number it must be a floating point number at all times. Using the definition of interactive numerals from §3.2, the program will use $N_{\text{int}}$ to evaluate the numeral *b*_*i*_ at each step *i* as the user enters it; furthermore, any user action that makes $N_{\text{int}}$ fail (i.e. fail to be a floating point value) will be ignored. As an interactive numeral is being constructed under this form of premature semantics, then at every stage it is interpreted as a meaningful number. But this means the program has prematurely implemented the user’s input as a number before they have finished interacting and confirmed it. In particular, it means that an erroneous sequence of interactions like 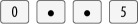 (and many others) cannot be processed correctly because the program has prematurely implemented the user’s input as a number and there is *no* floating point value that represents anything with two decimal points!

In other words, although the program *eventually* wants a number, in this case it has *prematurely* imposed the semantics of number instead of, for instance, string semantics (which can represent arbitrary keyed input from a user).

The example NaN given above is a symptom of premature semantics. Here, the computer has been programmed to prematurely use a number to represent the user’s input, but the user has attempted some interactive numeral action implemented on a number that the programmer has not correctly anticipated. This results in the program performing a non-numeric operation on what was prematurely (and incorrectly) programmed as a number value. The computer itself has responded with the number turning into an invalid number, namely NaN. Then the bad programming has failed to recognize this, and NaN (which is a technical term that is meaningless to normal people) is displayed directly to the user. In other words, displaying NaN proves the program has prematurely implemented something as a simple number (probably an IEEE float).

Shneiderman describes a common data quality problem [24]: a hospital was analysing the age distributions of patients and finding statistically significant differences, etc.; but Shniederman found patients who were 999 years old. Of course, clinicians do not always know a patient’s age but the program they used prematurely required a number (and nothing else). A patient’s age is certainly a number, but interactively there is a point before the number is correct or even known, as in this case. But premature semantics requires a number regardless. The clinicians thus have to find a workaround, such as entering 999, which is a number for the computer but it is not a number for the clinicians—it is an exception flag denoting ‘unknown.’ Unfortunately, 999 was an exception flag known to the users, but was not implemented by the programmers.

It is understandable that any such system needs some way of handling missing data but forcing a semantically correct number value here led to (perhaps well-intentioned) violations, a specific type of error [18] that can have serious consequences later. Indeed, this 999 violation worked every time, so it became standard practice. Yet it ruined data analysis, and probably disrupted hospital auditing and hence the day-to-day efficient operation of the hospital before Shneiderman uncovered the practice.

Now we recognize premature semantics as such, it explains a wide range of familiar problems. Web forms, for example, often require a user to fill in details they do not yet know: this is because the form implements the fields as simple values (numbers, dates, etc.), which premature semantics prevents from being left blank or partly filled in—a routine procedure that is very convenient and indeed trivial on paper forms.

A special case of premature semantics might be called **premature representation**—the representation is a consequence of an internal, premature, semantic choice. Many systems display a numeral even before anything has been entered, because the display is assumed to *always* display a number:

— Many interactive systems display 0 if the user has entered nothing: they then cannot distinguish a user who has entered nothing from one who has explicitly entered zero.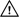 More generally, interactive systems displaying default values (such as 0) when the user has entered nothing makes it very difficult to distinguish a user entering a default value and *actually* doing nothing—not even recognizing ‘they’ have entered a value, which may therefore not be what they intended.
— Some systems display 0 to show that they are switched on, but this creates the ambiguity whether the user has started to interact or not. Instead, they could display patterns like 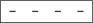 rapidly alternating with 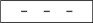 so they are obviously on and working.
— Many systems permanently display a decimal point [25], so if a user has entered nothing they will display 0. with a decimal point. This means that the user cannot tell whether they previously pressed just 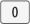 or 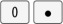: they cannot know from the display whether pressing 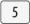 will change the number to either 0.5 or 5. This lack of predictability is a high price to pay for the ‘simplicity’ of always displaying a decimal point even when one has not been entered.

### 5.2. Feature interaction

**Feature interaction** is the name given to unanticipated interactions between system features [26]. Feature interaction is hard to anticipate because we focus so readily on individual features and think about them independently: it is much harder to think about features working together. In fact trying to think about two things at once *interferes* [27]: we do not do it very well and we tend to avoid doing it.

On calculators one feature is that we want users to be able to enter large numbers, and another feature is that the display screen can only show up to a fixed number of digits, typically 8 or so. The feature interaction here happens when a user keys in more than the allowed 8 digits—what should happen? Each feature separately makes sense, and in fact each feature is so straightforward and obvious it hardly needs thinking about at all. Yet, put together the two features conflict. Most calculators ignore the resulting feature interaction, with the result that the users will perform incorrect calculations without warning—and generally without noticing.

We want to allow users to make and correct errors, but that obviously desirable feature interacts with the equally obvious feature that only correct numbers are displayed. What should happen when a user keys in a syntactically incorrect number? How can it be corrected? This is another feature interaction.

Another feature interaction is the way negate works combined with the way delete works. The negate ( 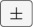 ) key changes the sign of a number and on most number entry systems it can be pressed at any time as a numeral is entered, in part because it is always represented as a prefixed minus sign whenever it is pressed. Thus, on the iOS calculator and many other devices with a change sign key, all of the following (pressed after 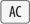 so they all start in the ‘ground state’): 
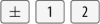
; 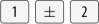; and 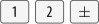 all result in −12 being displayed.

Delete seems simple, but is an interesting feature. In general, one would expect that a sequence of keystrokes …*k*_*i*−2_,*k*_*i*−1_,*k*_*i*_ followed by 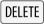 would be equivalent to …*k*_*i*−2_,*k*_*i*−1_. That is, the most recent keystroke *k*_*i*_ ‘disappears’ when 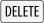 is pressed. Put into formal terminology, *k*_*i*_ 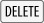 is the identity (for *k*_*i*_≠ 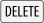 ), or it should be expected to be.

However, a feature interaction between change sign and delete arises. What happens when 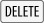 is pressed is unpredictable—it may or may not delete the sign prefix. The problem, of course, is that change sign key is implemented to put the negative sign at the left of the numeral, yet the delete key works on the right-most end of the numeral. After a change sign, the delete key cannot work both at the end and at the start of a numeral, and that causes an unfortunate feature interaction. A simple solution would be for the delete key to work on the *most recent* key pressed, not on the *right-most* key shown—but this is a little harder to implement.

The 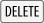 feature interaction is caused by premature semantics: treating an interactive numeral as a simple signed number, and then not having any representation of what the user has done so that 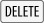 can be disambiguated.

### 5.3. Illegibility

If a numeral is ambiguous or unreadable (or inaudible if spoken, e.g. by synthesized speech) it is unreliable.

Legibility seems a self-evident criterion for a good interactive numeral, but as the Apple MacOS app example shows (figure 1*c*) it is easy to overlook. Other number entry systems routinely truncate numerals to fit displays, causing potential problems that are very hard to see. Elsewhere we have discussed legibility in more detail [25], and in particular, criticized poor choice of numeral fonts, such as seven segment displays.

Legibility ought to be an obvious requirement, and achieving it (particularly with today’s high-resolution displays and quality audio outputs) is completely unproblematic. Yet poor legibility continues to be a factor in accidents and other problems [25]—note that this reference gives brief details of a fatal plane crash caused by poor numeral legibility as well as many recommendations for improving numeral legibility more generally.

### 5.4. Ignoring error

Sooner or later, users make errors, and they may or may not be aware of their errors [17]. We have shown elsewhere [20] that if interactive numeral systems detect syntax errors (such as two decimal points) the effective error rate can be reduced; for instance, the ‘out by 10’ (the number entered is 10 times too large or too small) error rate can be halved.

In practice, interactive number entry systems rarely detect user error. We gave examples above of a user entering numbers that are too large: the results are wrong—yet detecting this error is trivial programming. In some systems, user error is ironically treated as a ‘feature’. For example, on the Graseby 3400 infusion pump (a drug delivery device) the decimal point is treated as a special case of 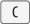: it deletes the decimal part of a number, so 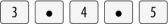 enters 3.5, whereas many other systems (like the Apple iOS and MacOS pane but not the MacOS app) will just ignore additional decimal points, even though they key-click, misleadingly confirming the keys are being processed normally.

Displaying NaN is a simple example of ignoring error. NaN is displayed when the program attempts to perform an invalid operation on a number: the hardware (or the virtual machine) has detected the error that the programmer has ignored (figure 1*b*).

Unlike the other Apple calculators, the MacOS app allows the user to enter arbitrarily large numbers, which as we explained above get displayed in a smaller and smaller font until they are unreadable. Continuing using the app eventually makes an erroneous calculation and displays NaN. Presumably, as each digit, say 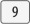, is pressed the interactive numeral processing calculates *d*′=10*d*+9, updating the displayed number *d* to *d*′, but eventually 10*d* or 10*d*+9 is too large for the underlying floating point representation to handle. A correct program would check *d* was in range before attempting an invalid calculation—even better, it would also check *d*′ would be displayed correctly (which none of the Apple calculators do). Even displaying the resulting NaN to the user is another ignored error.

All the three Apple calculators allow pasting in a number. For example, 1,2,3.4.5 becomes 123.4 on the iOS and pane calculators, but 123.45 on the app. None of the calculators detect the errors. Ignoring the commas ‘makes sense’ only because it is an easy way to handle correct numbers like 10,000. But the same reasoning also ignores problems with erroneous numerals like 10,00. Ignoring errors, including discarding digits and decimal points, is very unhelpful—users expect correct answers, not any old answers.

Many systems, however, do detect use error; for example, one of many studies using ‘smart pumps’ (interactive drug infusion devices with number range error detection) along with other interventions reduced error rates by 73% with paediatric patients [28]. Given the obvious benefits and effectiveness of error detection, it is, then, very surprising that interaction error is so widely ignored: it is clearly not a problem with reporting error as such (which is how so-called smart pumps work), but of bothering to program systems to detect errors during the process of interactive numeral entry.

Ignoring error is a special case of poor programming, which we discuss next.

### 5.5. Poor programming

Poor programming explains many problems.

We can describe the programming problem succinctly: user operations on numeral syntax define an abstract date type for data entry. The *continual* mapping of that abstract data type to computer number representations (e.g. floating point numbers) as the user enters and edits numbers becomes a mess if not programmed correctly.

**Figure 6.**
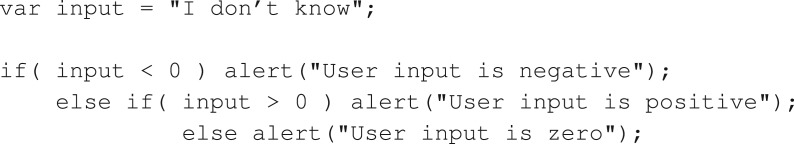
A user might enter the data ‘I don’t know’ into a numeric field on a web site programmed in JavaScript. JavaScript is a very popular web language but it is not type-checked. JavaScript will happily compare the non-numeric string the user has entered with the number zero, without any errors at compile time or run time. Since what the user entered is neither negative nor positive, the program will deduce it must be zero, which of course it is not. The error may then propagate through the program and cause further problems. In a type-checked language, strings and numbers are not comparable and a program like this would not compile, so the programmer would have to develop a ‘type correct’ program before it could be used.

Type checking helps detect inconsistencies *within* a program and helps improve program quality generally, but it does not specifically help improve interaction. To make the point, figure 6 illustrates problems that can arise without type checking. Clearly, some possible interaction design defects can be completely prevented by using a typed language, however type checking alone is not sufficient to correctly manage problems of the user entering ‘numerals’ that are not even parsable, as illustrated in the figure.

When interacting with programs, users can do anything, and a dependable interactive program has to respond coherently to all possible user interactions. Interestingly, neither the philosophies of static or dynamic type checking align well with this requirement: incorrect types cause incorrect programs to be rejected, and the programmer has to start again after fixing the errors. While that makes sense and is undeniably a very useful approach for dependable programming, by contrast, in user interface interaction rejecting the user’s input is rarely a good option—an interactive program has to keep running despite error, and it has to manage error as the user gradually repairs the input to something acceptable. Even if it may be a good idea for the user to ‘start again’ (e.g. when entering a quasi-numeral as an incorrect security code) the program cannot terminate.

Very often there are unhelpful compromises: the user can make an error, but their input will not be saved (or otherwise processed) until all the data are ‘correct’. This is essentially the type correct part of the program refusing to allow the user to continue interaction until all detected errors are fixed. If the user needs to pause while they decide how to proceed, many programs will further aggravate the situation by timing out and discarding the user’s partial input. Put in other words, the problem is the ‘correct’ program has required numbers prematurely, but the user interface is supporting interactive numerals—and the program is expecting the user only to save when all interactive numerals are well-formed numerals (and similarly for other types of data, not just numerals). As explained elsewhere in this paper (§5.1), this is a premature semantics.

Users learn how to enter numbers on systems and will acquire habits (such as shortcuts and specific ways to use the correction features). Thus users will internalize the ‘mess’ and if the mess is different on different systems, this will induce unnecessary errors (so-called transfer errors). The unnecessary variation, despite their very similar look and feel, between the three Apple calculators is a case in point.

Examples earlier showed large numbers may be accurately displayed when they are the result of a calculation, but not when the user enters them. Thus, although numbers as such are handled well, interactive numerals are not. Worse, there is usually no warning to the user that displayed values are incorrect when numbers entered by the user have been truncated.

The problems with large numbers probably happen because a standard, general-purpose, number output routine is used to display calculated values, but user-input numbers are displayed differently: a basic programming inconsistency. Considering the additional inconsistencies over cut and paste discussed above, it is likely that those calculators do not use standard text entry routines, but handle button presses in ad hoc ways.

We conclude that correct interactive numeral programming is harder than most people think—poor programming is deceptively easy. The cognitive effort to program well creates tunnel vision: that is, programmers make mistakes they are unaware of [29]. Modern software engineering techniques like formal methods and code review *must* be used to mitigate these predictable problems, and evaluation techniques *must* be used to evaluate whether designs meet their requirements when they are used.

The evidence presented in this paper suggests even major manufacturers are not using such techniques to help avoid problems. We would argue that any computer system programmed by people who do not use such techniques [30] should not be used for any critical application.


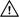
 Some readers of this paper have pointed out that some programming languages, such as Haskell and Swift (and more generally, many programming languages that have strong typing), have built-in mechanisms to help avoid premature semantics. However, the problems this paper has pointed out can be avoided in *any* programming language. The issue is not the programming language used, or the programming language that might have been used, or even whether the programmer uses data validation. The issue is the lack of knowledge about interactive numerals.

## 6. Conclusion

Interactive numerals are more complex than most programmers think, so they implement them simplistically. Even the world’s very best programmers are not immune. If programmers are unaware of premature semantics and feature interaction, they will make mistakes, which will then catch out users later. Understandably, users are unaware of these issues and they risk being misled, particularly in situations of error—precisely, then, at the very times when being misled is especially counter-productive.

This paper makes several important points:

(i) Our definition of interactive numerals is essentially equivalent to a high-level specification that programmers could implement. Variations from this specification would allow programmers to identify with clarity any design compromises, and so reason about the consequences of their compromises in their approach to interactive numerals.
(ii) The everyday practice of programming has not yet advanced to the point where interactive numerals are reliably implemented, nor to a point where users can reliably use them. We have developed some evaluation tools [21] that can rapidly highlight problems and help select safer designs, but formal methods should also be used to avoid problems in the first place.
(iii) Premature semantics is a concept describing a common type of defect in programming, and is particularly prevalent in poor interactive numeral implementations. Now named, it can be actively managed by programmers.
(iv) Problems with interactive numeral implementations mean that logs of interactive system usage may record what systems do but do not reliably record what was done by users. This hinders accurate understanding of any use problems, and may have legal repercussions: logs must not be naïvely used as evidence of what users have or have not done [31]—they only show what the system records *after* premature semantics has over-simplified it. Logs do not give any reliable insight into what users did (let alone why [31]), unless there is rigorous evidence of correct design and operation, including appropriate forensic procedures (e.g. digital signatures) to confirm log data is not contaminated by poor programming or cyber attacks.
(v) Interactive numerals can be discussed rigorously because numeral syntax and number semantics are familiar and are well-defined, precise concepts. It is, therefore, easy (if one asks the right questions) to show whether they are implemented correctly. There are, however, many other forms of interactive features that beg further study. For example, typing letters with accents (e.g. ï, é, ç, as well as keys that are not on some keyboards like æ, ß, ł, etc.) requires several keystrokes; what then should key sequences like 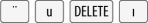 do? Indeed, the synchronization of the user model and the program’s semantic model at the keystroke level is a topic that has been excluded from classic research [32].

Being able to buy and use safe interactive numeral systems, let alone assure yourself they *are* as dependable as they seem, remains problematic. For the time being, in safety and mission critical areas users must compensate for poor design by adopting strategies (such as repeating calculations entered in different ways) to help detect error. It was beyond the scope of the present paper to discuss human factor mitigations, such as range validation and the user interface redisplaying numbers in contrasting numeral formats (e.g. spoken words) to help the user confirm that the number they intended to enter was indeed correctly entered.

With this paper, we hope we have clarified the fundamental role of interactive numerals in numerical interactive systems—that is, almost all interactive systems. From articulating the design and use errors around interactive numerals, our examples show that users, designer and procurers need to pay very close attention to their correct implementation. And implementations more consistent with our definitions are demonstrably possible. For instance, we can indicate contingent semantic correctness [10,33] or systematically avoid premature semantics [34–36]. But these are entirely new approaches for users and designers of number entry systems. They may bring new, different problems and there are most probably even better, more reliable, more analysable implementations. However, having identified now the conceptual target of interactive numerals, we are in a better position to work towards the engineering of more dependable interactive systems. The extra effort needed has a very high leverage because, for many applications, getting interactive numerals right will help millions of users over the lifetime of the improved products.

## Footnotes

1 ‘Radix point’ is the generic term for decimal point, without regard for the base of the numeral. This paper generally uses the term ‘decimal point’ because of its familiarity, but without implying any loss of generality.

2 Excel’s (v. 15.30 for OSX) PRODUCT calculates the product of a set of numbers. Curiously, if the set *only* contains 5.5 kg, etc., the product is zero, but if there are ‘conventional’ values as well in the set, like 32, then the PRODUCT operation treats 5.5 kg as 1.0. The documentation for PRODUCT is incorrect too: ‘You can also perform the same operation by using the multiply (*) mathematical operator; for example, =A1*A2.’ But * does not implement the same operation, as it gives #VALUE! when PRODUCT behaves as described above. This suggests that Microsoft has lost track of the semantics of their interactive numerals.

3 Similar problems are ubiquitous across many devices and applications. In this paper, we present some initial examples from Apple calculators, though figure 2 shows problems are not limited to Apple. Apple have a leading reputation for high quality user interfaces, and also develop across several distinct and widely-available platforms. We provide version numbers so results can be easily reproduced.

4 E and e are commonly used interchangeably in decimal numerals in programming languages to mean power of 10, so programmers may already be comfortable with the notation, but this does not excuse carrying over a programming notation into a general-purpose user interface notation, especially when it so obviously creates knock-on problems for the user. Most users are not programmers.

5 Obviously the decimal value 0.1 can be represented using structured binary values, such as rationals (e.g. as two binary numbers, representing 1 divided by 10) or as binary coded decimal, and in many other ways.

## Data accessibility

All experimental data and results are fully described in the paper.
